# Specific formulas for preterm infants, how and when

**DOI:** 10.1186/1824-7288-41-S1-A46

**Published:** 2015-09-24

**Authors:** Antonio Alberto Zuppa, Piero Catenazzi, Riccardo Riccardi, Costantino Romagnoli

**Affiliations:** 1 Department of Pediatrics, Division of Neonatology, Catholic University of the Sacred Heart, Rome, Italy

## 

Both ESPGHAN (2010) and AAP (2012), stated that “all preterm infants should receive human milk” for the many short-term and long termbenefits[1,2].

All kinds of breast milk (fresh by own mother or pastourized by donor) for preterm should be fortified, to gain the recommended requirements.

In case of its absence the only alternative is represented by the formulas for preterm infants (PTF).

It is not yet definitively established the ideal PTF composition, particularly for ELBW infants. Table 1 shows the main recommendations for nutrients[1-4].

A study compared the use of a soy-based formula (with calcium, phosphorus and vitamin D), with a PTF. Infants taking soy showed lower growth, levels of protein and albumin[5]. ESPGHAN in 2006 concluded that soy-based formulas should not be used in premature infants[6].

The use of hydrolyzed formulas has not shown a preventive role on cow's milk protein allergy, it has proven helpful in improving food tolerance (acceleration of the intestinal transit time and faster achievement of full enteral feeding), but it has a reduced nutritional value, (especially protein intake)[7-11].

A recent study evaluated the usefulness of a thickened formula in reducing apnea of prematurity GERD-related. The authors conclude that these formulas are not effective in the reduced number of apneas GERD-related[12].

In recent reviews post-discharge formulas does not seem to provide benefits, especially for the heterogeneity of the studies[3,13]. They may be useful for infants with GA <33 weeks, particularly those <30 weeks, with growth at discharge below the 10th percentile (the ESPGHAN recommended their use up to 40 weeks, and for a further 12 weeks if necessary) [3].

Studies about GOS and FOS showed an increase of bifidobacteria in the stool, a reduction in their viscosity and an acceleration of intestinal transit time, resulting in an easier achievement of full enteral feeding[14,15]. It is also assumed a role in the prevention of NEC and LOS. Even though they have proven their beneficial role, further studies are needed to establish the type and dose[16].

Several RCTs and recent reviews have shown a benefit of prebiotics in reducing NEC andin the achievement of full enteral feeding[17-19]. Further studies are needed to establish dose, strains and routes of administration[1]. 

Lactoferrin, both human and bovine, seems to have a significant role as a protective agent against NEC and LOS[20-23].

The available data do not allow to recommend formula supplementation with these substances with functional properties.

**Table 1:** Recommended intakes for macro and micronutrients [1-4]

Nutrient	Per 100 kcal	Recommendations
Energy, Kcal	-	A reasonable range of energy intake for healthy growing preterm infants with adequate protein intake is 110 to 135 kcal*kg^-1^*day^-1^

Protein, g (VLBW)	3,3 - 3,6	Protein supply needs to compensate for the accumulated protein deficit observed in almost all small preterm infants.The quality of the provided protein may interfere with the recommended intake because the infant does not require proteins but requires specific aminoacids. Whey predominant protein with reduced glycomacropeptide and α-actalbumin enrichment could be used to optimize the amino acid profile.
	
Protein, g (ELBW)	3,6 - 4,1	

Carbohydrates, g	10,5 - 12	According to the relatively reduced intestinal lactase activity, the lactose content could be relatively reduced and replaced by glucose polymers with the characteristic of maintaining the low osmolality of the formulas.

Lipids, g	4,4 - 6	In order to improve fat absorption, an important quota of fat could be given as medium-chain triglycerides with a maximum of 30–40% of lipid content.

Calcium, mg	110 - 130	The calcium to phosphorus ratio (1,5 – 2) may be an important determinant of calcium absorption and retention.
	
Phosphate, mg	55 - 80	

Iron, g	1,7 - 2,7	Iron is essential for brain development, and prevention of iron deficiency is important. Prophylactic enteral iron supplementation (given as a separate iron supplement) should be started at 2 to 6 weeks of age (2–4 weeks in extremely-low-birth- weight infants) and should be continued after discharge, at least until 6 to 12 months of age depending on diet.
